# OFraMP: a fragment-based tool to facilitate the parametrization of large molecules

**DOI:** 10.1007/s10822-023-00511-7

**Published:** 2023-06-13

**Authors:** Martin Stroet, Bertrand Caron, Martin S. Engler, Jimi van der Woning, Aude Kauffmann, Marc van Dijk, Mohammed El-Kebir, Koen M. Visscher, Josef Holownia, Callum Macfarlane, Brian J. Bennion, Svetlana Gelpi-Dominguez, Felice C. Lightstone, Tijs van der Storm, Daan P. Geerke, Alan E. Mark, Gunnar W. Klau

**Affiliations:** 1 School of Chemistry & Molecular Biosciences, The University of Queensland, Brisbane, QLD 4072 Australia; 2 Centrum Wiskunde & Informatica, Science Park 123, 1098 XG Amsterdam, The Netherlands; 3 Algorithmic Bioinformatics, Heinrich Heine University Düsseldorf, Universitätsstr. 1, 40225 Düsseldorf, Germany; 4 Department of Chemistry and Pharmaceutical Sciences, Amsterdam Institute of Molecular and Life Sciences (AIMMS), Vrije Universiteit Amsterdam, De Boelelaan 1108, 1081 HZ Amsterdam, the Netherlands; 5 Department of Computer Science, University of Illinois at Urbana-Champaign, Urbana, IL 61801 USA; 6 Biosciences and Biotechnology Division, Lawrence Livermore National Laboratory, 7000 East Ave, Livermore, CA 94552 USA; 7 Department of Chemistry, University of Connecticut, 55 North Eagleville Road, Storrs, CT 06269 USA; 8 Faculty of Science and Engineering, University of Groningen, Nijenborgh 4, 9747 AG Groningen, The Netherlands

**Keywords:** Molecular fragments, Force fields, Automated topology builder, Partial charges, Drugs, Dendrimer, Molecular simulation

## Abstract

An Online tool for Fragment-based Molecule Parametrization (OFraMP) is described. OFraMP is a web application for assigning atomic interaction parameters to large molecules by matching sub-fragments within the target molecule to equivalent sub-fragments within the Automated Topology Builder (ATB, atb.uq.edu.au) database. OFraMP identifies and compares alternative molecular fragments from the ATB database, which contains over 890,000 pre-parameterized molecules, using a novel hierarchical matching procedure. Atoms are considered within the context of an extended local environment (buffer region) with the degree of similarity between an atom in the target molecule and that in the proposed match controlled by varying the size of the buffer region. Adjacent matching atoms are combined into progressively larger matched sub-structures. The user then selects the most appropriate match. OFraMP also allows users to manually alter interaction parameters and automates the submission of missing substructures to the ATB in order to generate parameters for atoms in environments not represented in the existing database. The utility of OFraMP is illustrated using the anti-cancer agent paclitaxel and a dendrimer used in organic semiconductor devices.

**Graphical abstract:**

OFraMP applied to paclitaxel (ATB ID 35922).
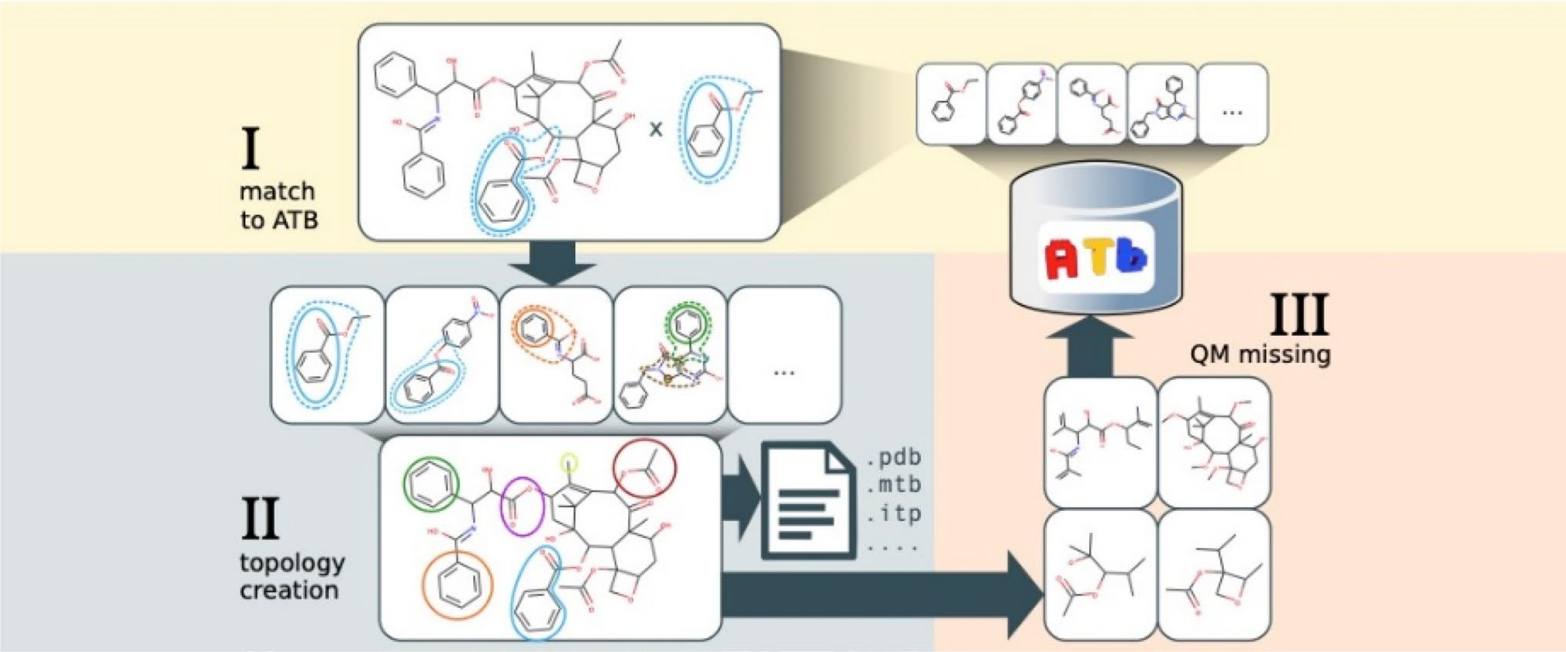

**Supplementary Information:**

The online version contains supplementary material available at 10.1007/s10822-023-00511-7.

## Introduction

The simulation of molecular systems at an atomic or near atomic level is playing an ever-increasing role in fields ranging from computational drug design to the analysis of functional materials. The utility of molecular simulations is intimately linked to the reliability and consistency of the parameters used to describe the interatomic interactions. While highly optimized parameters have been developed for systems ranging from simple alkanes to biomolecules such as proteins, lipids or nucleic acids, these represent just a small fraction of the chemical space of interest. Over the last two decades, various automated parametrization tools have been developed to facilitate the parametrization of novel molecules e.g., RED [1], GAAMP [2], PRODRG [3], antechamber [4], CGenFF [5, 6], ffTK [7], LigParGen [8], OpenMM [9] and the Automated Topology Builder (ATB) [10–12]. These topology builders assign force field parameters (such as point charges, bond lengths, bond angles and torsion angles) using a variety of approaches. Most commonly parameters are obtained by fitting to the results of quantum–mechanical (QM) calculations or using empirical rules to recognize specific chemical moieties and assigning parameters to these moieties based on a set of previously parameterized reference molecules. The aim of all these builders is to produce force fields capable of describing the structural and thermodynamic properties of arbitrary molecules with high accuracy. While such approaches are effective for relatively small organic molecules (< 50 atoms), the extension of current schemes to larger molecules that cannot be represented as a combination of simple sub-units (e.g., biopolymers such as proteins), remains a major challenge. This is because simple rule-based approaches lack the precision to describe the local chemical environment of atoms within an arbitrary molecule and approaches that rely on QM calculations become infeasible as the size of the molecule increases. A high-level theory such CCSD(T) can be applied to small organic molecules (*e.g.,* the anti-inflammatory agent ibuprofen, C_13_H_18_O_2_). However, for larger drug molecules such as the anti-cancer agent paclitaxel (C_47_H_51_NO_14_, CHEMBL428647 [13]) even geometry optimization at a modest level of theory such as density functional theory (DFT) in combination with the B3LYP/6-31G* functional and basis set (as used currently by the ATB [10–12]) involves significant computational cost. In addition, the algorithms used to infer the value of partial charges and bonded parameters become less reliable. This is due to the increased ambiguity in the fitting of partial charges to the electron density and in the projection of the QM Hessian [14] onto specific degrees of freedom (used in many routines to assign bond and angle force constants) for larger molecules.

One solution to this problem is to use a fragment-based approach. That is to assign parameters for a target molecule based on a series of smaller molecules parameterized in isolation. This is the approach historically used to manually parametrize biomolecules in force fields such as GROMOS [15], AMBER [16] or CHARMM [17]. Specifically, a series of fragments (building blocks) is defined and parametrized based on a small set of reference compounds (e.g., analogs of amino acid side chains). This approach is appropriate and effective in the case of linear biopolymers such as DNA, RNA, peptides/proteins, and simple polysaccharides in which the individual units are linked in a consistent manner (phosphodiester bonds for DNA and RNA, amide bonds for peptides).

Using a fragment-based approach for general molecules, such as required in drug design and material science applications, is much more complex. The chemical space of interest is large and even though the basic chemical moieties found in drug-like molecules may be similar, the local chemical environment in which these moieties are found can vary significantly. This means that finding the most appropriate reference molecule within an existing database can be challenging. It may be that only one molecule in a large database is relevant, or there may be hundreds of potential reference molecules, each varying slightly. Finally, while the individual sub-units of (bio)polymers (e.g., amino acids, nucleotides and sugars) can be treated as independent and linked together in a consistent manner, this is not true for moieties in other molecules. Even in molecules that have common substructures, these substructures will be connected via a variety of intermediate atoms. In such cases, rather than treating the molecule as composed of independent substructures, a better approach would be to represent the molecule as a series of overlapping sub-fragments, with neighboring groups joined by a common substructure.

**Fig. 1:**
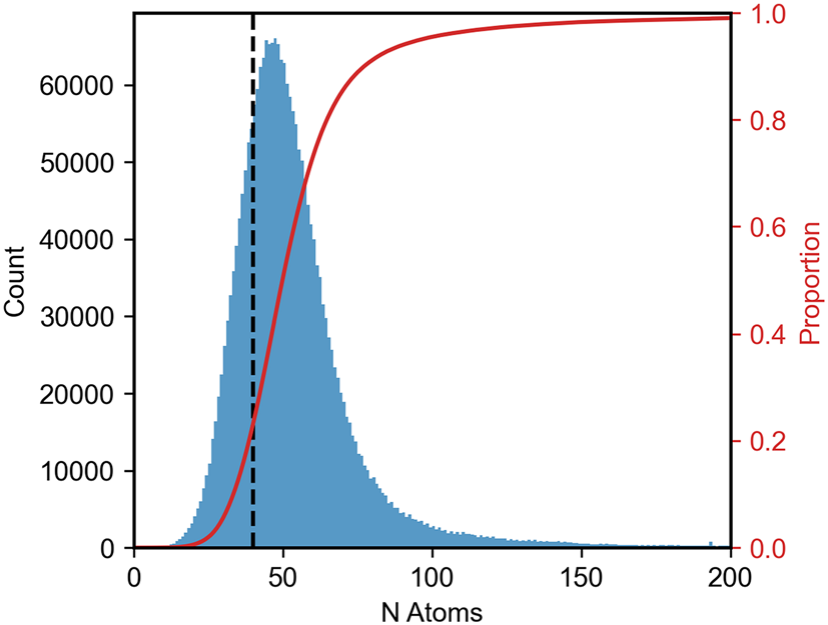
A histogram showing the number of molecules containing a given number of atoms within the ChEMBL 32 database [20] (blue bars) and the cumulative proportion (red line). Currently, all molecules up to 40 atoms (dashed line) have been parametrized using the Automated Topology Builder [10–12] (ATB)

There are multiple approaches that can be used to address this problem. If the chemical diversity within the set of molecules of interest is limited, one can attempt to define a set of reference fragments from which all other molecules can be constructed. This is the approach used in the program MATCH, developed by Yesselman et al. [18] for use in conjunction with the CHARMM family of force fields. A series of reference fragments that characterize an atom in a specific chemical environment were defined which could then be used to assign appropriate atom types. These atom type fragments were defined by the authors using a combination of “expert knowledge” and automated procedures [18]. The atoms in a query molecule are assigned to a specific type within a given force field using a graph-based tree matching algorithm. MATCH was also used to develop what the authors referred to as “bond charge increment rules” used to infer charges for a novel molecule based on connectivity [18]. CherryPicker [19] uses a similar graph-based approach focusing on matching fragments from an existing library of building blocks. For example, CherryPicker can be used to assign parameters for molecules with the same chemical functionality and connectivity as peptides. The limitation of the approach used in both these programs is the implicit assumption that the molecular fragments in the reference set stipulated by the developers can represent all query molecules appropriately. This assumption is questionable for applications such as drug design, as the number of molecules for which parameters are needed is very large. As illustrated in Fig. 1, the ChEMBL [20] database (version 32) of bioactive compounds contains approximately 2.3 million molecules, with approximately half of these having more than 50 atoms. The ZINC [21] database of commercially available compounds currently contains more than 230 million entities. Given the number of molecules involved, defining appropriate reference molecules for all possible chemical environments contained within these databases is challenging. MATCH deals with novel environments by interpolation between parameters or extrapolation into new regions of parameter space. However, there is also the question of which fragment(s) in the existing database best match those in the query molecule and the (fixed) rules used to address any potential conflict. For example, does the program average the charges from alternative matching fragments or select a set of default values [22].

Here we present an Online tool for Fragment-based Molecule Parametrization (OFraMP) which avoids many of the limitations of the procedures outlined above. OFraMP identifies sub-structures (fragments) within a given query molecule that match sub-structures in molecules that have been parametrized previously. The algorithm identifies all possible matching fragments using a hierarchical approach by considering atoms embedded within a specific local chemical environment defined in terms of a user specified neighborhood size (buffer region). Matched fragments are then ranked by the degree of overlap (the number of identical atoms within the matching sub-structures). In contrast to current alternatives, OFraMP uses a semi-automated selection procedure in which the algorithm presents possible matches to the user who then chooses the most appropriate reference molecule based on their understanding of the specific system. OFraMP also includes a simple and robust semi-automated tool for combining overlapping fragments to obtain parameters for novel molecules, again allowing the user to select between a range of options. Finally, if there are no appropriate fragments within the existing database to represent a part of the query molecule, OFraMP will generate one or more molecules covering the missing part of chemical space expanding the existing database.

The fragment identification routines in OFraMP can in principle be used as a stand-alone program where the user provides a library of parametrized fragments. However, OFraMP has been primarily developed for use in conjunction with the Automated Topology Builder (ATB) force field development tool [10]. The ATB has both the capacity to generate new parameters for novel molecules as well as a database of more than 890,000 pre-parametrized compounds. This includes 25% of the ChEMBL [20] database (all molecules up to 40 atoms, see Fig. 1), 80% of the ligands found within structures of the Protein Data Bank and all molecules that have been involved in a clinical trial [23]. The ATB has been extensively validated with respect to its ability to reproduce the conformational and solvation properties of a wide range of molecules [12]. For a validation set of 685 molecules the average unsigned error between free energy of hydration values calculated using ATB (3.0) parameters and experiment is 3.8 kJ·mol^−1^. The slope of the line of best fit is 1.00, the intercept − 1.0 kJ·mol^−1^, and the R^2^ 0.90. This demonstrated that in terms of the prediction of solvation properties, the ATB parameters equaled, or outperformed, alternatives including GAFF [24–26], GAMMP [26], LigParGen [27] and OPLS3 [28]. Linking OFraMP to the ATB allows parameters to be assigned to molecules that are too large to be processed efficiently using QM methods given current computational limits. Specifically, OFraMP allows for partial atomic charges assigned to molecules already present in the ATB to be transferred to equivalent sub-structures in larger molecules while maintaining the fidelity of all other terms. In this use case OFraMP provides a mechanism to extend the ATB, it does not represent a new force field description.

Linking OFraMP and the ATB achieves two aims. First, it provides access to the partial charges assigned to atoms in hundreds of thousands of molecules all of which have been parameterized in a consistent manner. Second, it provides access to the algorithms used by the ATB to assign atom types (van der Waals parameters) and bonded terms. ATB atom types are assigned based on the hybridization state of the atom (inferred from its connectivity with its nearest neighbors) and the local chemical environment (inferred from the types of atoms to which it is connected, coupled with the partial charge assignment). Bonded terms are assigned by matching the local sub-structure in the molecule to a set of predefined structural templates for which reference values have been calculated. If an appropriate match to a structural template cannot be found, new bonded parameters are generated based on a QM Hessian (< 40 atoms). [10]

As atom types and bonded parameters in the ATB (and most empirical force fields) depend primarily on local interactions, they are readily transferable between molecules. In contrast, the distribution of charges is dependent on the three-dimensional geometry of the molecule and long-range interactions between groups of atoms. Consequently, partial charges are more difficult to transfer between molecules. In the case of the ATB, for molecules containing < 50 atoms, partial charges are fitted to reproduce electrostatic potential (ESP) for individual molecules calculated at the DFT (B3LYP/6-31G*) level of theory. For molecules containing between 50 and 500 atoms the partial charges are calculated using semi-empirical approaches (e.g., at the AM1 level of theory with MOPAC charge assignments). In the case of molecules containing between 500 and 1000 atoms (the current maximum molecule size) no charge assignment is attempted.

The combination of OFraMP (with its ability to identify and match sub-fragments) and the ATB (with its large existing database of molecules with QM-derived charges) provides users with an efficient and robust means to assign ESP derived charges (B3LYP/6-31G*) to atoms in molecules, which due to their size can only be treated using less accurate semi-empirical QM approaches or parameterized using group based charge models. Note, in the implementation used in this work the assignment of atom types and bonded terms is performed by the ATB independently of OFraMP. However, as the ATB assigns atom types and bonded terms based on local sub-structures, identical results would be obtained if the atom types and bonded terms were extracted from the matched fragments. The only difference would be that a larger *shell size* would be needed to ensure consistent assignments for the dihedral terms in some cases.

OFraMP is described in detail below. Two worked examples are also provided to illustrate how OFraMP can bridge the gap between molecules that can be parameterized automatically using the ATB and larger molecules where fragment matching and input from the user enables parameters of comparable quality to be assigned.

## Molecular graph matching

Within OFraMP, molecules are represented as graphs where the nodes correspond to atoms labeled by an atom type and the edges to covalent bonds between these atoms. The key element of the matching algorithm used in OFraMP is that an atom in one molecule is only considered equivalent to an atom in another molecule if the atoms in question, together with all neighboring atoms within the declared buffer region, are of the same type and have the same connectivity (number of nearest neighbors). The buffer region used in OFraMP is defined in terms of the number of intervening bonds. In the case of the default *shell size* of 3, all atoms connected by 3 bonds or less are considered neighbors of the atom in question.

To find all possible matching fragments within a pair of molecular graphs, the problem to be solved is Enumerating all Maximal Common Fragments (*k*-MCF–E). It can be shown that the *k*-MCF–E problem is a generalization of enumerating all maximal Common Connected Induced Subgraphs (CCIS) (MCCIS–E) [29]. Note, there is a fundamental difference between enumerating all maximal CCIS (MCCIS–E) and the well-known problem of finding the maximum (largest) CCIS. Many exact algorithms and heuristics have been proposed finding the maximum CCIS, especially in the context of molecular graphs [30–32]. Due to its combinatorial nature, finding all maximal CCIS (MCCIS–E) is much more challenging. For OFraMP the MCCIS–E algorithm of Koch [33] was adapted to *k*-MCF–E. We have shown previously that when combined with additional data reduction techniques, this algorithm for solving *k*-MCF–E is highly efficient even for large molecular graphs [29], allowing databases containing 100,000s of molecules to be screened within seconds. A detailed description of the algorithm used is provided as Supplementary Information (SI).

**Fig. 2:**
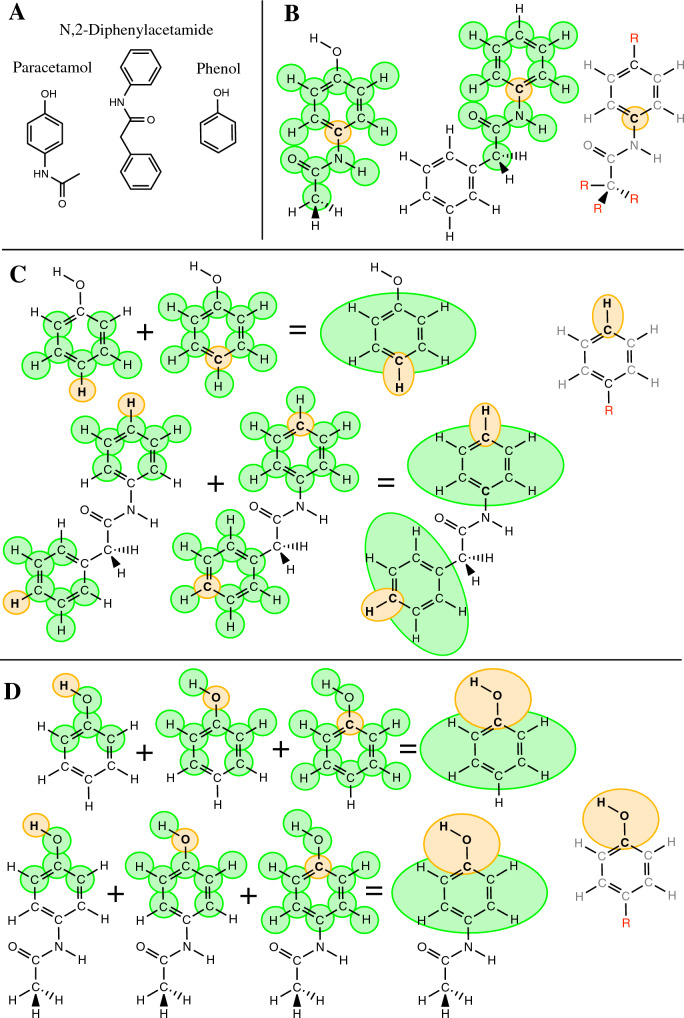
An illustration of different *k*-maximal common fragments between paracetamol, *N*,2-Diphenylacetamide and phenol showing the identification of fragments with 1, 2 and 3 matched atoms determined using a *shell size k* = 3. **A** Structural formulas of paracetamol, *N*,2-Diphenylacetamide and phenol. **B** A common fragment between paracetamol and *N*,2-Diphenylacetamide. The common carbon atom (light orange) surrounded by buffer region (green) spanning 3 bonds (a *shell size* of *k* = 3). The resulting single atom fragment and associated Common Connected Induced Subgraph (CCIS) is shown to the right. R represents an arbitrary group. **C** Common fragments between *N*,2-Diphenylacetamide and phenol. First column: a hydrogen atom (light orange) in an equivalent environment in the two molecules (*k* = 3). Second column: a carbon atom (light orange) in an equivalent environment in the two molecules (*k* = 3). Third column: Given they are adjacent the hydrogen and carbon that are common between the two molecules can be combined into a two-atom common fragment. The atoms in the buffer region are indicated (green). The resulting two-atom fragment and associated CCIS is shown to the right. **D** Common fragments between phenol and paracetamol. First column: a hydrogen common to both molecules. Second column: an oxygen common to both molecules. Third column: a carbon common to both molecules. Fourth column: being adjacent the hydrogen, oxygen and carbon can be combined to form a three-atom common fragment. The final three-atom fragment and associated CCIS is shown to the right

Figure 2 provides a series of examples of the common molecular substructures for paracetamol, *N*,2-Diphenylacetamide, and phenol (Fig. 2A) with 1, 2 and 3 matching atoms (Fig. 2B–D, respectively) determined using a *shell size* of 3. Figure 2B shows an example of a single matching atom fragment found on both paracetamol and *N*,2-Diphenylacetamide. On the left is shown the ring carbon in question in paracetamol (orange) surrounded by atoms in the buffer region (green). In the middle is shown the equivalent atom in *N*,2-Diphenylacetamide together with atoms in its buffer region. The complete fragment is shown on the right. Note, all atoms that form part of the substructure have been assigned the same atom type and have the same connectivity, but only the central atom is considered equivalent in the two molecules. There are no restrictions on the nature of the groups outside the buffer region indicated by *R*. Figure 2C shows common fragments between *N*,2-Diphenylacetamide and phenol. Figure 2D common fragments between phenol and paracetamol.

## OFraMP within the ATB

OFraMP is intended to facilitate the parameterization of molecules that are too large to be treated efficiently or robustly using DFT QM methods. In principle, all interaction parameters could be extracted from a set the fragments matched using only information related to element type and connectivity. However, in the ATB implementation of OFraMP, information is provided in the form of an initial or template topology generated using a 3-dimesional coordinate file. This greatly simplifies subsequent steps and helps minimize the size of the buffer required to achieve appropriate matches. An appropriate set of coordinates on which to base the initial template topology can be generated using the inbuilt JSME molecule builder [34], from an initial 3D structure provided in a PDB (Protein Data Bank) format [35], or generated from a SMILES [36] (Simplified Molecular Input Line Entry Speciation) string from which a 3D structure is generated within the ATB using RDKit [37]. These initial topologies contain a preliminary list of all atom types, bonds, angles, dihedrals and exclusions. Note, template generation is the initial stage of the ATB parameterization pipeline. Academic users can generate template topologies for molecules containing up to 1000 atoms without restriction. For molecules containing up to 500 atoms, the geometry of the molecule is optimized, and charges assigned using semi-empirical approaches. By default, the ATB only performs DFT calculations if the molecule contains less than 50 atoms.

Once the initial topology has been generated, a new entry is automatically added to the ATB database. OFraMP is accessed by following the *Fragment-Based Parameterization with OFraMP* link on the corresponding ATB molecule page (atb.uq.edu.au). The buffer region used to determine whether two atoms are embedded within identical local environment is determined by the user defined parameter *shell size,* which is passed to OFraMP. As noted above, by default, the *shell size* is set to 3. The meaning of *shell size* is illustrated in Fig. 2. The sub-structure identification calculations (described above) scale approximately linearly with the number of atoms in the query molecule. For the current database size of 890,000 molecules, OFraMP queries take between 4 min (50 atoms) and 30 min (1000 atoms). Currently, the results of a query are stored for 7 days during which time sub-structure matches can be retrieved within seconds.

## OFraMP workflow

**Fig. 3:**
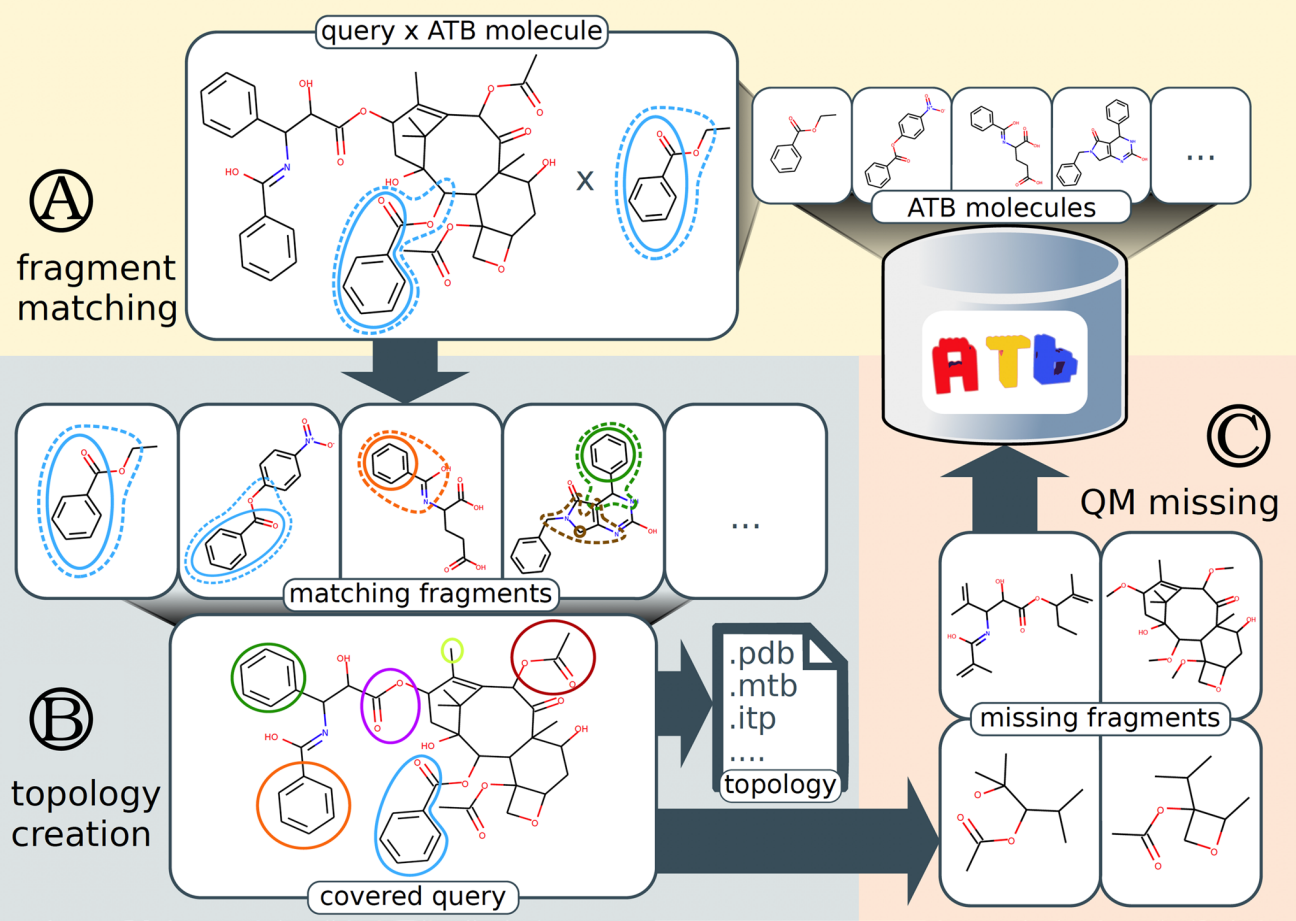
General overview of the OFraMP workflow applied to paclitaxel (ATB ID 35922). **A** OFraMP matches the query molecule to parametrized molecules in the ATB and returns all matching fragments. **B** If the molecule can be fully represented using existing fragments in the ATB database, the user iteratively selects fragments to cover the query molecule and generates a topology file; else **C** OFraMP generates one or more molecules covering the missing fragments (for which no parameters are available) which the user can send to the ATB to be parametrized and added to the database

Figure 3 gives an overview of the OFraMP workflow as applied to paclitaxel (ATB ID 35922: https://atb.uq.edu.au/molecule.py?molid=35922). Upon loading a molecule, OFraMP identifies common sub-structures (fragments) between the query molecule and all molecules in the ATB database. The user can then select fragments from the database that best match the atoms in the query molecule. If parts of the query molecule are not currently represented in the database, the user can choose to assign the charge parameters by hand or opt to send these missing fragments to the ATB to be parametrized automatically. Note, processing of the molecule containing a missing fragment can take from hours to days depending on the molecule size and the load on the ATB server at the time. In addition, as the results from the initial OFraMP query are stored (*cached*) for 7 days, if you return to the system within this time the results from the initial search must be deleted (using the *Delete cached OFraMP run* button) for the newly processed missing fragment to be identified as a match.

Once all atoms have been assigned partial charges, the full set of partial charges can be sent to the ATB (using the *Send charges to ATB* button). These charges can be accessed under the under the *Fragment-Based Charges* tab on the given molecules page within the ATB. Coordinate and topology files incorporating these charges can then be generated in various formats.

## User interface

**Fig. 4:**
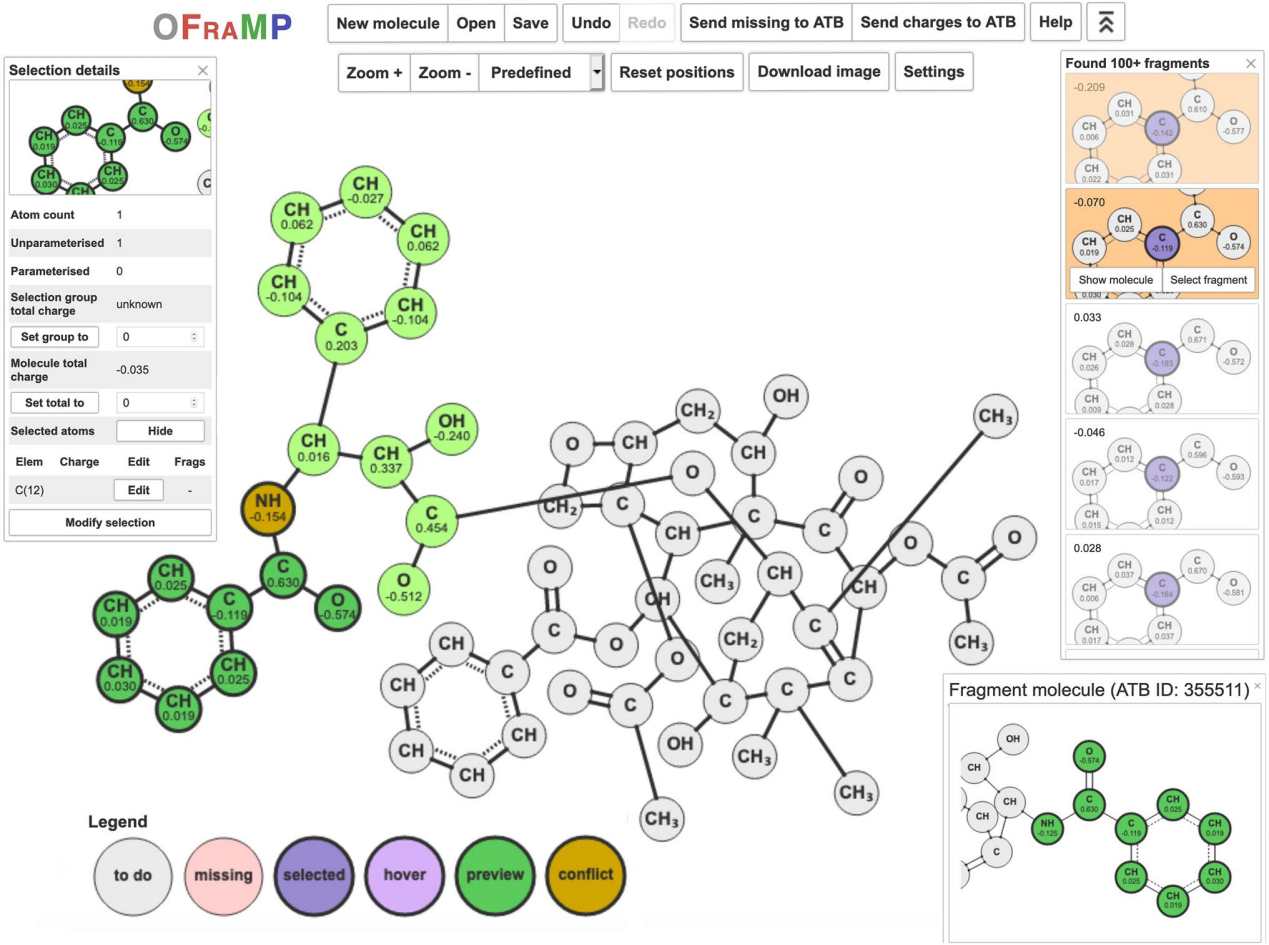
The OFraMP web interface. The query molecule (paclitaxel, ATB ID 35922) is displayed in the middle. Atoms matched to at least one molecule in the database but not yet assigned (grey). Parametrized atoms with assigned point charges (light green). Atoms showing an unresolved conflict (ochre). Left panel: information pertaining to the atom(s) currently selected (dark green). Upper right panel: a list of fragments in the ATB matching the selected atom(s). Fragments that overlap with parameterized atoms (orange). Lower right panel: the reference molecule containing the selected fragment

Figure 4 shows the OFraMP graphical user interface. After a structure is loaded, the user is presented with a 2-dimensional representation of the molecule. For ease of visualization, a united-atom representation is used for CH_1_, CH_2_ or CH_3_ groups by default. Parametrization is, however, always based on an all-atom representation. The display of non-polar hydrogens can be modified under the *Settings* menu*.* A range of parameters that control how the 2-dimensional representation of the molecule is generated and displayed can also be varied (e.g., the radii of the nodes, font size etc*.*).

OFraMP starts by determining if each atom in the query molecule matches an existing molecule in the ATB database given the *shell size* selected. Matched atoms are shown in grey. Atoms for which no matches could be found are colored pink. These missing atoms can either be sent to the ATB for parameterization (described below) or the user can assign charges manually.

For atoms represented in the existing database, the user proceeds by selecting one or more atoms from the query molecule using the graphical interface. Information on the atoms selected is shown in the *Selection details* panel on the left-hand side of the window. A list of matching fragments is presented on the right-hand side, sorted by the extent of overlap with the query molecule (number of atoms). If the cursor is moved over one of the matching fragments, atoms in the query molecule that form part of the fragment are previewed in dark green. The total charge on each fragment is also displayed. The charge can be used to help identify the most appropriate fragment, for example, a fragment from a molecule with the same formal charge.

After clicking on a *Found* fragment, the user can elect to *Show molecule*. This displays the molecule within the ATB database containing the corresponding fragment in the same graph representation as the query molecule so that the user can to compare the two chemical environments. Clicking *Select fragment*, transfers the charges from the fragment to the corresponding atoms in the query molecule. Atoms to which charges have been assigned are shown in light-green. The user then selects another atom, and the assignment process is repeated until all atoms have been assigned a charge. Note, in many cases a new fragment may partly overlap with fragments chosen previously. (Sub-)fragments that overlap with fragments previously selected are shown in ochre. Although the shell surrounding a given fragment is the same, there can still be differences in the charges assigned to particular atoms due to long-range effects and uncertainties in the ESP fitted charges leading to conflicts. The magnitude of these differences will largely depend on the choice of *shell size* and whether the molecules that contain the matched fragments selected from the ATB database represent similar environments (e.g., do the molecules have the same net charge). For these overlapping atoms (ochre), the user is asked to either: (a) select one of the alternative partial charges, (b) average the values, or (c) manually provide a new value. Atoms which cannot be mapped to an existing fragment (pink) can be parametrized manually by selecting a given atom and setting the charge in the *Selection details* panel.

Because the partial charges assigned to individual atoms most probably have come from independent molecules, the formal (total) charge of a molecule parametrized using OFraMP can deviate from the required integer value. The difference between the target charge and the sum of assigned charges is referred to as the residual charge. Once charges are assigned to all atoms, an interface is provided displaying the residual charge. The user can opt to allow OFraMP to correct the overall charge by subtracting from each atom the residual charge divided by the number of atoms in the query molecule. Alternatively, the user may alter the charges on specific atoms to eliminate the residual charge. If appropriate fragments have been selected, the residual charge should be small. If the residual charge is large, a fragment from a molecule carrying an inappropriate net charge may have been selected. Once all atoms have been assigned a charge, the user can send the result to the ATB. Note, the *total charge* on the molecule is also given in the *Selection details* panel on the left which can be displayed by selecting any atom on the query molecule.

## Output

Atomic charge distributions obtained using OFraMP and which have been sent to the ATB are assigned a unique identifier (OFraMP ID) and accessible via the *Fragment-Based Charges* tab on each molecule page. The ATB algorithm is then able to generate topology files using the OFraMP-assigned charges. Note that the ATB refines the assignment of particular van der Waals parameters based on the partial charge of the atom [12]. The rational for this stems from the fact that van der Waals interactions reflect the distribution of electron density around different atoms. An sp3 hybridized carbon with a large positive partial charge has, by definition, less electron density than an sp3 hybridized carbon with a large negative partial charge. The final values for the bonded terms (i.e., bonds, angles, dihedral angles) are generated using the same algorithms used to assign these terms in all molecules in the ATB. Note, in some cases the query molecule will contain a novel chemical environment for which no appropriate bond or angle types are available. In such cases, any additional bond and angle parameters will be added to the ATB parameter files based on an analysis of the QM Hessian. This will occur automatically as the missing fragment is processed by the ATB. The final all-atom or united-atom topology can be provided in the following formats: GROMOS [38], GROMACS [39], X-plor [40], CNS [41], CIF [35], LAMMPS [42], and APBS. [43]

## Missing fragments

In many cases, there will be atoms within the query molecule for which no matching fragment exists in the current ATB database. OFraMP groups these “missing” atoms into fragments which can then be automatically submitted to the ATB for processing using the button *Send missing to ATB*. In this way, the chemical space represented in the database will be extended, allowing the query molecule to be fully covered. It will also ensure that any novel bonds, angles or dihedrals are incorporated into the force field description.

The molecule that is sent to the ATB for processing must not only include the fragment representing the missing atoms itself but also the local chemical environment as defined by the *shell size*. A molecule required to parameterize even a few missing atoms can be large. To ensure molecules incorporating a missing fragment can be processed by the ATB, in some cases a missing fragment must itself be split into sub-fragments. This is achieved by progressively dividing the molecule containing the missing atoms until each section is below a specified limit, currently set to target molecules with 30–40 atoms. There is an exception to this size limit in cases where the minimum fragment would result in ring structures being broken. Molecules submitted for processing by OFraMP can be monitored via the ATB *Existing Molecules* interface by selecting the *Processing Molecules* checkbox. Note, the ATB performs a series of calculations to progressively improve the parameterization of a given molecule beginning with geometry optimization at a semi-empirical level and finally the calculation of the QM Hessian at the DFT (B3LYP/6-31G*) level of theory to identify and parameterize novel bonded parameters [10]. The calculation of the QM Hessian for a molecule containing 40 atoms can take several days on the available resources.

## Fragment capping

Fragments identified by the approach described above are chemically incomplete (uncapped) i.e., some atoms will have an incomplete valence structure. These must be capped for the molecule containing the missing fragment to be processable by the ATB. The capping is chosen so as not to introduce a net charge, polar groups or alter the nature of ring structures. To achieve this, a series of capping groups for each chemical element have been defined. For example, an aliphatic carbon atom is capped with either 1, 2 or 3 hydrogens, depending on its hybridization state. The valence structure of the fragment is completed by finding an optimal combination of capping fragments for each uncapped end (atom). This optimization is performed using an Integer Linear Program (ILP), which is well suited to such combinatorial problems. This allows large systems, containing many uncapped atoms, to be processed rapidly. This capping algorithm is described in [44].

## Illustrative examples

To illustrate the capability of OFraMP to parametrize complex molecules in a consistent and robust manner, two examples are presented: the parametrization of the widely used anti-cancer agent paclitaxel [45] and the parametrization of a dendrimer [46] used in the development of organic semiconductor devices. In both cases, a *shell size* of 3 was used.

Paclitaxel is a challenging case consisting of 113 atoms and containing a complex sub-structure involving multiple fused rings (Figs. 3, 4). To illustrate the degree of consistency in the charges that can be expected when using OFraMP, 5 alternative starting atoms were chosen. These atoms were well separated to ensure the starting fragment was different in each case. The initial fragment was chosen from the five largest fragments that overlapped with the query molecule. The next fragment was obtained by selecting another unparametrized atom and choosing the largest matching fragment that had no overlapping atoms with the first fragment from the five largest fragments. Again, combinations of fragments used in previous attempts were avoided. This was repeated until charges were assigned to all atoms. In cases where overlapping core regions of neighboring fragments were unavoidable, the conflicts in the partial charges were resolved by averaging the alternative values. Finally, to ensure that the net charge on the molecule matched the target value (zero), a correction to remove the residual charge was applied evenly over all atoms. The reason to generate charge distributions in this way is to illustrate the degree of potential variation in the charges. In the ideal case, if the *shell size* was sufficiently large, the residual charge should be zero and the partial charges obtained from the five independent parametrizations should be identical. For a *shell size* of 3, the absolute value of the residual charge varied between 0.028 *e* and 0.445 *e*, with an average of 0.169 *e*. Of the 113 atoms in paclitaxel the standard deviation of the assigned partial charges was greater than 0.1 *e* for just 13 atoms. All but one of these 13 atoms was a buried carbon. It was greater than 0.2 *e* for just four atoms. The largest variation in charge (0.515 *e*) was observed for a buried ester oxygen. In four assignments the charge for this atom was between − 0.214 and − 0.393 *e*, while for the other assignment it was − 0.729 *e*, suggesting that the conformation of this group in the reference molecule chosen was significantly different to the conformation in the reference molecules chosen during the other four assignments. The corresponding charge sets can be viewed on the ATB under the *Fragment-Based Charges* tab on the molecule page (https://atb.uq.edu.au/molecule.py?molid=35922). A specific set of charges can be selected by choosing the corresponding OFraMP_ID. Note, there are currently 13 conformers of paclitaxel in the ATB each with a separate entry and corresponding ATB ID. The fragment-based charges generated using OFraMP are currently only displayed on the molecule page of the specific conformer used during the generation process. However, the identification of matching fragments is independent of the conformer chosen.

**Fig. 5:**
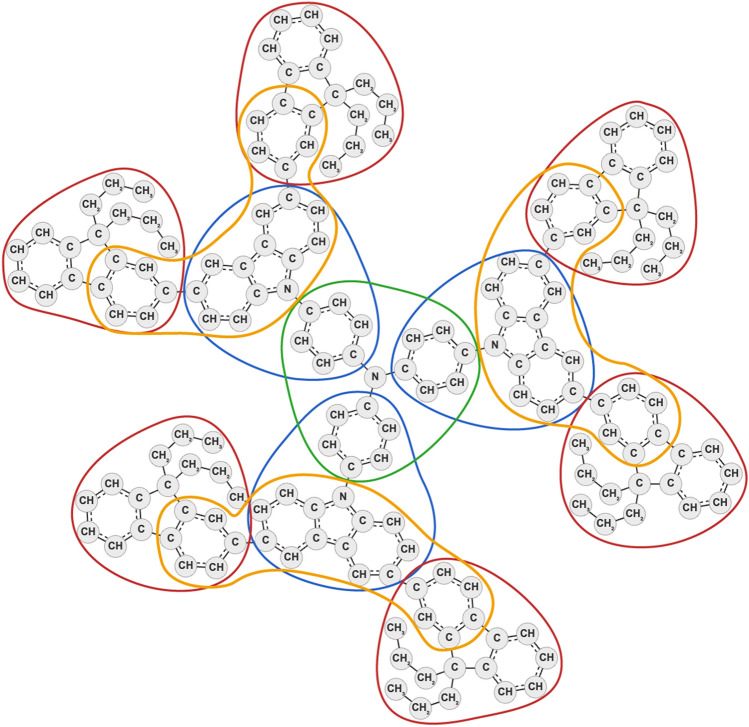
A 328-atom dendrimeric structure in the OFraMP graph representation. The four independent fragments used to represent the complete tree are highlighted

In the second application, OFraMP was used to parametrize the 328-atom dendrimer (https://atb.uq.edu.au/molecule.py?molid=704360) shown in Fig. 5. In this case the complete dendrimer tree could be covered with just five fragments. While smaller fragments with non-overlapping atoms could have been selected, the use of larger (overlapping) fragments in this case minimizes any potential discontinuity between fragments and increases the effective buffer region. If the fragments have been chosen appropriately, any differences in the partial charges of the overlapping atoms will be slight (a small fraction of a unit charge). In this example the differences were resolved by using the inbuilt averaging tool. While a challenge for many topology builders, the parameterization of branched and dendritic structures is very straightforward using OFraMP. The corresponding topology file can be obtained from the ATB as part of the *Fragment-Based Charges* tab on the molecule page (https://atb.uq.edu.au/molecule.py?molid=704360). The user can also choose whether to symmetrize the charges in the molecule before the topology and corresponding coordinate files are generated. The set of charges generated using the fragments shown in Fig. 5 correspond to OfraMP_ID = 91.

## Conclusion and perspectives

OfraMP leverages existing calculations to parameterize novel molecules using a semi-automated approach. The resources invested in the parameterization of molecules within the ATB database have been significant. This was made possible by the development of automated workflows and access to large scale computational facilities within Australia and the USA. A significant proportion of the molecules in the database were processed using facilities at the Lawrence Livermore National Laboratory (LLNL). For this, a workflow was established as a part of other ongoing studies whereby researchers at LLNL could remotely request a molecule from the ATB, process this molecule locally and then upload the output to the ATB for parameter assignment, resulting in the molecule becoming part of the ATB database. Three Linux clusters maintained at LLNL were incorporated into this workflow, Borax and Quartz (36 CPUs/node 128 GB memory/node) as well as Catalyst (24 CPUs/node, 128 GB memory/node). This allowed approximately 190,000 compounds containing between 20 and 35 atoms to be processed over an 18-month period.

While the number of molecules processed via this pipeline was substantial, it corresponded to less than 10% of the ChEMBL [17] database. Furthermore, the molecules involved were comparatively small considering that more than half of the molecules in ChEMBL contain more than 50 atoms (Fig. 1) and the DFT computations scale approximately as the number of valence electrons to the third power. Even with the access and advantage of using high performance computing at such large-scale facilities, the cost of processing all molecules in the ChEMBL database at the DFT (B3LYP/6-31G*) level of theory is still prohibitive.

Currently, the ATB contains over 890,000 molecules, including all molecules in the ChEMBL database with 40 atoms or less. An examination of 1250 randomly chosen ChEMBL molecules containing between 49 and 51 atoms yet to be added to the ATB database, found that for a *shell size* of 3, approximately 94% of the atoms could be matched with atoms in the existing database, with 40% of the molecules fully covered. The distribution of atom coverage is provided as SI (Figure S1). While it is expected that the larger the *shell size* the better the match, a *shell size* of 3 already leads to very consistent parameters. For instance, the N-methylbenzamide fragment (with a *shell size* of 3) in paclitaxel is found in 56 different ATB molecules. The standard deviation of the partial charges on the atoms within the core of the fragment are all below 0.1 *e*. The fact that 40% of the ChEMBL molecules sampled are fully covered and that a majority of the remaining molecules are missing 5 atoms or less (Fig. S1) indicates that the ATB already covers a significant proportion of chemical space represented in the ChEMBL database. However, this result also highlights the challenge when using approaches that rely on user assigned fragments such as MATCH. Even using a database containing in excess of 890,000 related molecules, over 60% of the trial molecules contained atoms in novel chemical environments. In principle, each case would require a new reference fragment to be defined by hand. The power of OFraMP is that these missing fragments are detected and then parameterized automatically. In this way the use of OFraMP will lead to the systematic expansion of the chemical space represented within the ATB database.

As illustrated in the case of paclitaxel and the example dendrimer, OFraMP provides an efficient means to parametrize large molecules in a consistent and robust manner, leveraging both the processing machinery and the very large number of molecules that have been parameterized to a high level within the ATB. As the ATB database continues to grow and the range of molecules expands with time, the utility of OFraMP in terms of the accuracy of the parameters provided and its ability to process more complex molecules will only increase. As noted above, when used in conjunction with the ATB, OFraMP is only required to assist with the assignment of partial charges. This is because the assignment of atom types and bonded interactions in the ATB is itself based on matching local sub-structure.

It is important to note that the protocols outlined in this work and implemented in OFraMP simply provide a systematic and consistent means to assist a user in extending an existing force field to larger and potentially more complex molecules. The underlying character of the existing force field is therefore retained. Currently, ATB parameters are based on a fixed charge model. However, the machinery incorporated into OFraMP could equally be applied to a polarizable model. ATB charges are derived by fitting to the electrostatic potential obtained after the geometry optimization of the molecules at the DFT (B3LYP/6-31G*) level of theory. By default, the structural optimization and ESP calculation is performed in the presence of an implicit continuum solvent with a relative dielectric of 80 [10–12]. Given this, the default ATB charges can be considered to have been tuned for use in the condensed phase (water). The ATB employs a more sophisticated charge model than some other force fields. ESP fits are performed using symmetry constraints and with a much higher fidelity than that suggested by earlier works [10–12]. This reduces (but does not eliminate) numerical instabilities during the fitting of charges and thus the need for constraints to be applied on the charges of buried atoms as in RESP [47]. The additional computational cost of obtaining these charges makes the availability of tools such as OFraMP particularly important. Indeed, one reviewer questioned why DFT and ESP charge fitting was used as opposed to a more approximate approach such as AM1-BCC charges [48]. Certainly, semi-empirical methods such as AM1 can be applied to larger molecules than DFT, reducing the need for approaches such as OFraMP. However, AM1 derived charges perform poorly when predicting experimental properties such as solvation free energy and must be adjusted using empirical correction terms. The widely used bond charge corrections (BCCs) to AM1 charges were initially derived by fitting to ESP charges at the HF/6-31G* level of theory using a training set of over 2700 molecules. At the time this was claimed to be sufficient to sample most organic functional groups and combinations thereof. For a test set of small bi-molecular complexes, the root mean squared difference in the interaction energy calculated using AM1-BCC charges and HF/6-31G* was found to be in the order of 1 kcal mole^−1^ [48]. While this level of accuracy might be sufficient in many applications the approach has limitations. First, the ability to systematically improve the charges is limited given that the base level of theory (AM1) is fixed. Second, the approach depends on corrections that have been fitted using a specific training set. As is evident from the work outlined, even considering a database of over 800,000 reference molecules combined with a modest shell-size of just 3, atoms in novel chemical environments are still being found in more than 50% of new molecules extracted from the ChEMBL database. In contrast, the charge model used within the ATB contains no empirical derived parameters and is readily extendable to larger molecules using OFraMP. Using ESP charges directly without fitted corrections or scaling factors, the ATB parameters equaled, or outperformed, alternatives including GAFF [24–26], GAMMP [26], LigParGen [27] and OPLS3 [28] in the prediction of hydration free energies. Most importantly, there is a clear pathway by which the ATB model might be further improved. For example, the DFT functional used is being migrated from B3LYP to ωB97X [49] and alternative solvation models are being tested. This is of course computationally demanding and requires a level of resources not available to all. However, rather than relying on a low-cost approximate approach with multiple researchers performing essentially identical calculations independently, we feel that making the results of higher-level calculations freely available to all in the academic community is not only a more efficient use of publicly supported computational facilities, but ultimately promotes better science.

Finally, OFraMP is distributed under an open-source (MIT) license. Although OFraMP was developed in conjunction with the ATB, the core methodology could be adapted to work with other topology generators and/or molecular databases. A version of the code most suited to be used independently of the ATB is available on GitHub [50].

## Disclaimer

This document was prepared as an account of work sponsored by an agency of the United States government. Neither the United States government nor Lawrence Livermore National Security, LLC, nor any of their employees makes any warranty, expressed or implied, or assumes any legal liability or responsibility for the accuracy, completeness, or usefulness of any information, apparatus, product, or process disclosed, or represents that its use would not infringe privately owned rights. Reference herein to any specific commercial product, process, or service by trade name, trademark, manufacturer, or otherwise does not necessarily constitute or imply its endorsement, recommendation, or favoring by the United States government or Lawrence Livermore National Security, LLC. The views and opinions of authors expressed herein do not necessarily state or reflect those of the United States government or Lawrence Livermore National.

## Supplementary Information

Below is the link to the electronic supplementary material.

Supplementary file1 (PDF 540 KB)
