# Supplementary material for: OFraMP: a fragment-based tool to facilitate the parametrization of large molecules
Source: J Comput Aided Mol Des. 2023 Jun 13;37(8):357–71. doi: 10.1007/s10822-023-00511-7 (PMC10315351; doi:10.1007/s10822-023-00511-7)
Supplement: Supplementary file 1 — Supplementary file1 (PDF 540 KB) [file 10822_2023_511_MOESM1_ESM.pdf]

OFraMP: A Fragment-Based Tool to Facilitate the Parametrization of Large Molecules

Martin Stroet,[†] Bertrand Caron,[†] Martin S. Engler,[‡,¶] Jimi van der Woning,[‡] Aude Kauffmann,[†] Marc van Dijk,[§] Mohammed El-Kebir,[I] Koen M. Visscher,[§] Josef Holownia,[†] Callum Macfarlane,[†] Brian J. Bennion,[⊥] Svetlana Gelpi-Dominguez,[#] Felice C. Lightstone,[⊥] Tijs van der Storm,[‡,@] Daan P. Geerke,[§] Alan E. Mark,[†] and Gunnar W. Klau[¶]

[†]*School of Chemistry & Molecular Biosciences, The University of Queensland, Brisbane, Queensland 4072, Australia*

[‡]*Centrum Wiskunde & Informatica, Science Park 123, 1098 XG Amsterdam, The Netherlands*

[¶]*Algorithmic Bioinformatics, Heinrich Heine University Düsseldorf, Universitätsstr. 1, 40225 Düsseldorf, Germany*

[§]*AIMMS Division of Molecular Toxicology, Department of Chemistry and Pharmaceutical Sciences, Faculty of Science, Vrije Universiteit Amsterdam, De Boelelaan 1108, 1081 HZ Amsterdam, the Netherlands*

[I]*Department of Computer Science, University of Illinois at Urbana-Champaign, Urbana, IL 61801, USA*

[⊥]*Biosciences and Biotechnology Division, Lawrence Livermore National Laboratory, 7000 East Ave, Livermore, CA 94552, United States*

[#]*University of Connecticut, Department of Chemistry, 55 North Eagleville Road, Storrs, CT 06269, United States*

[@]*Faculty of Science and Engineering, University of Groningen, Nijenborgh 4, 9747 AG Groningen, The Netherlands*

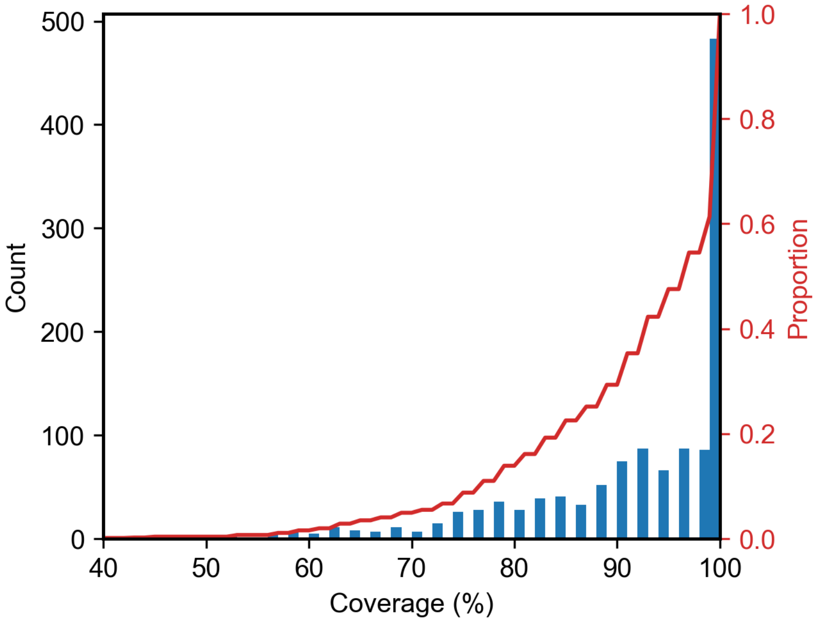

Figure S1. The distribution of atom coverage (blue) produced by OFraMP in combination with the 850,000 molecules currently in the ATB for 1250 query molecules with 49 to 51 atoms selected at random from the ChEMBL database. The cumulative total (red) shows the proportion of molecules with a particular coverage (%) or less.

Identification and partitioning of missing atoms into parameterisable fragments: Algorithmic details

General definitions

A molecule is modeled as a *molecular graph*, a simple graph $G = (V, E)$ whose nodes and edges correspond to atoms and bonds, respectively. Nodes are labeled by their atom type $t : V \to \Sigma$, where Σ is the set of all atom types. It is expected that the parameters assigned to atoms will vary depending on the presence of nearby substituents. To account for this, atoms are considered in the context of their neighborhood:

Definition 1 (k-neighborhood)**.** *Given a graph $G = (V, E)$, let the k-*neighborhood *$N^k(u)$ of a node $u \in V$ be the set of all nodes $v \in V$ for which a path $(u, \ldots, v)$ of length $\leq k$ exists.*

Partitioning of missing atoms into small fragments

If it is not possible to match all atoms to a fragment in the existing database, OFraMP collects all unparameterisable nodes $V' \subseteq V$ within a query molecule and returns the connected components of the subgraph induced by the union of k-neighborhoods $\bigcup_{u \in V'} N^k(u)$ for parameterisation by the ATB. However, by default the ATB imposes a maximum number of nodes $M = 50$ for parameter assignment based on higher level quantum calculations. Therefore, OFraMP needs to partition molecular graphs which exceed this size restriction into sub-graphs. Our partitioning strategy is the following: First, the number of partitions should be minimised. Second, partitions should overlap and ring systems in the graph should be retained in at least one partition. Partitioning is achieved by examining whether the molecular graph is biconnected. A graph is *biconnected*, if it remains connected after removing any one of its nodes. A *biconnected component* (or *block*) of a graph is a maximal biconnected subgraph. The smallest block is an edge (also called *bridge*). It is easy to see that a ring system is a block in a molecular graph. If a graph is connected and has two

blocks, they share one node, the *cut node* (Fig. S2a).

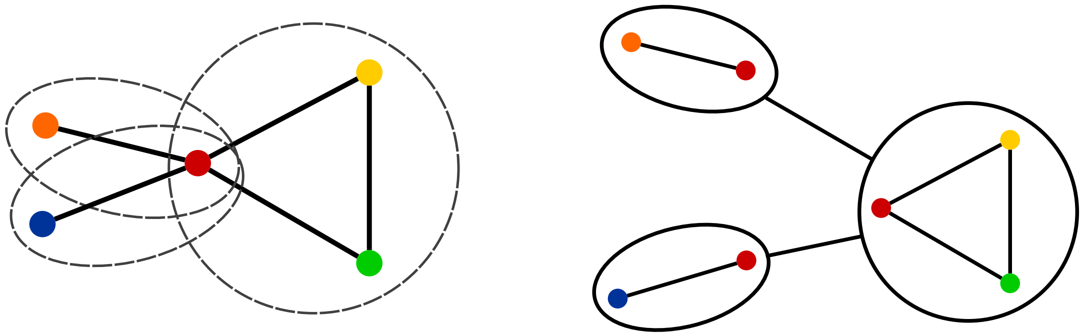

(a) Blocks and bridges (dashed lines) of a small example graph.

(b) BB-tree with block and bridge nodes of the same small example graph.

Figure S2: (a) Blocks and bridges in a simple graph and its (b) corresponding BB-tree. The red node is a cut node and shared between all three blocks or bridges.

Based on the idea of the block-and-bridge-preserving operator introduced by Horváth *et al.*,[S1] we define the *k-s-block-and-bridge-preserving (BBP) partitioning problem* as:

Problem 1 (k-s-BBP-Partitioning)**.** *Given $k \geq 0$, $s \geq 1$ and a molecular graph $G = (V, E)$, select a set of subsets $P = \{V' \mid V' \subseteq V\}$, such that*

(i) every node $v \in V$ is in at least one partition $\bigcup_{V' \in P} V' = V$,

(ii) all partitions are smaller than the maximum size $\forall V' \in P : |V'| \leq s$,

(iii) the number of partitions $|P|$ is minimal,

(iv) every block in G is in at least one partition $V' \in P$, and

(v) partitions overlap, such that if $u \in V$ is a cut node and $u \in V'$, then $N^k(u) \in V'$.

The *block-and-bridge tree* or *BB-tree* is a tree $T = (V^T, E^T, f)$ with $f : V^T \rightarrow \{v \in V\}$ which maps tree nodes to blocks in the graph $G = (V, E)$ (Fig. S2b).[S1] Nodes in the BB-tree are connected by an edge, if they share a cut node.

To solve the k-s-BBP-Partitioning problem, we partition the molecular graph recursively (Alg. 1). If the current partition is too large and has more than one block, we partition the

graph into two smaller partitions V_1, V_2 using its BB-tree representation. We then increase the overlap of the partitions by adding the k-neighborhoods of the cut node w.

Algorithm 1: k-s-BBP-Partitioning(G, k, s)

Input: Molecular graph $G = (V, E)$, shell size k, maximum number of nodes s
Output: k-s-BP-partitions of G

```
if |V| ≤ s then
    return G                                    // partition is small enough
else
    T = (V^T, E^T, f) ← BB-tree of G
    if |V^T| = 1 then
        return G            // one biconnected component, cannot partition
    else
        e = (u, v) ← BB-k-BalancedCut(T, G, k)               // cut edge
        if e = NULL then
            return G                   // partitions cannot become smaller
        T_1 = (V_1^T, E_1^T, f) ← first connected component of T' = (V^T, E^T \ e, f)
        T_2 = (V_2^T, E_2^T, f) ← second connected component of T' = (V^T, E^T \ e, f)
        {w} ← f(u) ∩ f(v)                                     // cut node
        G_1 = (V_1, E_1) ← subgraph of G induced by ⋃_{v'∈V_1^T} f(v') ∪ N^k(w)
        G_2 = (V_2, E_2) ← subgraph of G induced by ⋃_{v'∈V_2^T} f(v') ∪ N^k(w)
        k-s-BBP-Partitioning(G_1, k, s)
        k-s-BBP-Partitioning(G_2, k, s)
```

To determine how to partition the graph, we find a balanced cut edge e in the BB-tree $T = (V^T, E^T, f)$ of $G = (V, E)$:

Problem 2 (k-BB-BalancedCut)**.** *Given $k \geq 0$, a molecular graph $G = (V, E)$, and its BB-tree $T = (V^T, E^T, f)$, find a cut edge $e \in E^T$, such that for the resulting partitions $V_1, V_2 \subset V$*

(i) the size difference $||V_1| - |V_2||$ is minimal and

(ii) partitions overlap, such that if $u \in V$ is a shared cut node in V_1 and V_2, then $N^k(u) \in V_1$ and $N^k(u) \in V_2$.

We solve the k-BB-BalancedCut problem using a simple tree traversal algorithm (Alg. 2). We iterate all nodes $u \in V^T$ of the BB-tree sorted in ascending order by the number of their

unprocessed neighbors and mark u as processed. Because we iterate a tree, there is always either a node with exactly one unprocessed neighbor (starting with the leafs) or, in the final iteration, the root node with no unprocessed neighbors. The sizes of partitions that would result by cutting the edge $e = (u, v)$ of u and its unprocessed neighbor v are computed. The size of the current partition w_u corresponds to the size of union of nodes in G mapped by the nodes of the subtree of T rooted at u. The size of the other partition w_r corresponds to the rest of G. As it is required that the partitions overlap, the sizes of the k-neigborhoods of the cut node of $f(u)$ and $f(v)$ are added to determine if the partitions are still smaller than G. Finally, we return the edge resulting in the minimal size difference between both partitions.

Algorithm 2: k-BB-BalancedCut(T, G, k)

Input: BB-tree $T = (V^T, E^T, f)$ of graph $G = (V, E)$, shell size k
Output: $e \in E^T$ cut edge

$Q \leftarrow$ PriorityQueue
$d_{min} \leftarrow \infty$
$e \leftarrow$ NULL
foreach $v \in V$ **do**
    Q.insert(deg(v), v)   `// nodes ordered by degree`
    $N^{\Diamond}(v) \leftarrow \{u \mid (u, v) \in E\}$ `// unprocessed neighbors of` v
    $w_\Sigma(v) \leftarrow 0$ `// number of nodes represented by subtree rooted at` v
while *not Q.empty()* **do**
    $u \leftarrow Q$.extractMin()
    foreach $v \in N^{\Diamond}(u)$ **do**
        $\{x\} \leftarrow f(u) \cap f(v)$ `// cut node`
        $w_u \leftarrow w_\Sigma(u) + |f(u)| + |N^k(x) \setminus f(u)|$ `// nodes of` G `in subtree of` u
        $w_r \leftarrow (|V| - w_\Sigma(u) - |f(u)| + 1) + |N^k(x) \setminus f(v)|$ `// nodes of` G `in rest`
        if $w_u < |V|$ *and* $w_r < |V|$ *and* $|w_u - w_r| < d_{min}$ **then**
            $d_{min} \leftarrow |w_u - w_r|$
            $e \leftarrow (u, v)$
        $w_\Sigma(v) \leftarrow w_\Sigma(v) + |f(u)| + w_\Sigma(u) - 1$
        $N^{\Diamond}(v) \leftarrow N^{\Diamond}(v) \setminus u$
        Q.decreaseKey(v)

The k-neighborhoods of all $v \in V$ can be computed in a preprocessing step using a breadth-first-search which in the worst case has a complexity of $O(|V| + |E|)$ for each node.

Overall, the time complexity for the preprocessing is $O(|V| \cdot (|V| + |E|))$. The algorithm visits each edge of the BB-tree exactly once and the number of edges in T is bounded by the size of G with at most $O(|E|)$ edges. Extracting the minimum from Q requires $O(log(|E|))$ time using a Fibonacci-Heap, with all other operations on Q performing in constant time. Overall, the time complexity of finding the optimal cut edge is $O(|E| \cdot log(|E|))$.

Applying the k-s-BBP-Partitioning algorithm to a large unparameterisable fragment of paclitaxel with $k = 1$ and $s = 50$ results in five overlapping partitions with the ring system contained in one of the partitions (Fig. S3).

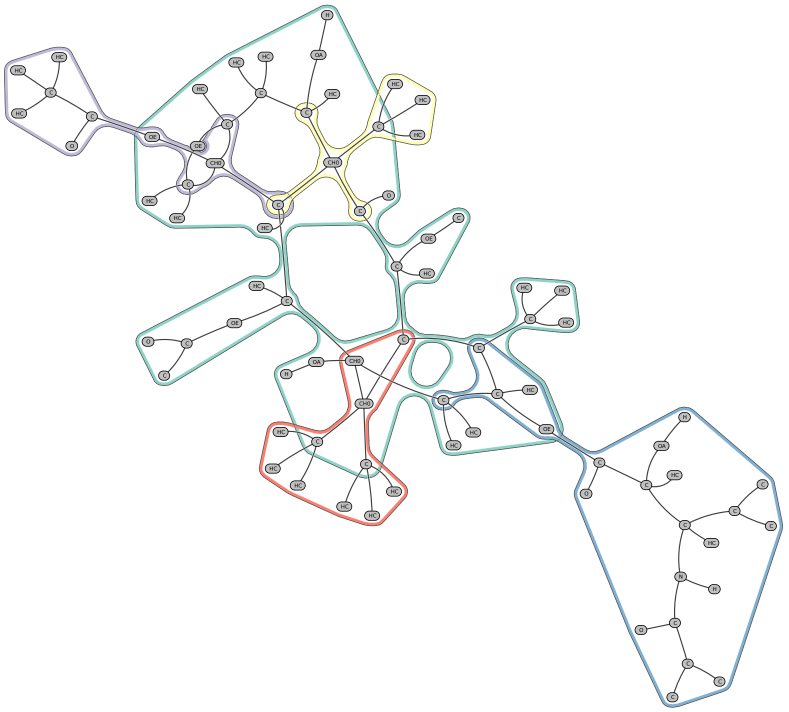

Figure S3: k-s-BBP-Partitioning of a large unparameterisable fragment of paclitaxel with $k = 1$ and $s = 50$.

References

(S1) Horváth, T.; Ramon, J.; Wrobel, S. Frequent subgraph mining in outerplanar graphs. Proceedings of the 12th ACM SIGKDD international conference on Knowledge discovery and data mining. 2006; pp 197–206.
